# A robust *in vitro* model for trans-lymphatic endothelial migration

**DOI:** 10.1038/s41598-017-01575-w

**Published:** 2017-05-09

**Authors:** Yanbao Xiong, C. Colin Brinkman, Konrad S Famulski, Emmanuel F. Mongodin, Colin J. Lord, Keli L. Hippen, Bruce R. Blazar, Jonathan S. Bromberg

**Affiliations:** 1 Center for Vascular and Inflammatory Diseases, University of Maryland School of Medicine, Baltimore, USA; 2 Alberta Transplant Applied Genomics Centre, University of Alberta, Edmonton, Canada; 3 Institute for Genome Sciences, University of Maryland School of Medicine, Baltimore, USA; 4 University of Minnesota Cancer Center and the Department of Pediatrics, Division of Blood and Marrow Transplantation, University of Minnesota, Minneapolis, 55455 USA; 5 Department of Microbiology and Immunology, University of Maryland School of Medicine, Baltimore, USA; 6 Department of Surgery, University of Maryland School of Medicine, Baltimore, Maryland 21201 USA

## Abstract

Trans-endothelial migration (TEM) is essential for leukocyte circulation. While much is known about trans-blood endothelial migration, far less is known about trans-lymphatic endothelial migration. We established an *in vitro* system to evaluate lymphatic TEM for various cell types across primary mouse and human lymphatic endothelial cells (LEC), and validated the model for the murine LEC cell line SVEC4-10. T cells exhibited enhanced unidirectional migration from the basal (abluminal) to the apical (luminal) surface across LEC, whereas for blood endothelial cells (BEC) they migrated similarly in both directions. This preferential, vectorial migration was chemotactic toward many different chemoattractants and dose-dependent. Stromal protein fibers, interstitial type fluid flow, distribution of chemokines in the stromal layer, and inflammatory cytokines influenced LEC phenotype and leukocyte TEM. Activated and memory CD4 T cells, macrophages, and dendritic cell (DC) showed chemoattractantΔdriven vectorial migration, while CD8 T cell migration across LEC was not. The system was further validated for studying cancer cell transmigration across lymphatic endothelium. This model for lymphatic TEM for various migrating and endothelial cell types possesses the capacity to be high-throughput, highly reproducible and integrate the complexities of lymphatic biology, stromal variability, chemoattractant distribution, and fluid flow.

## Introduction

Trans-endothelial migration (TEM) is an essential process for leukocyte circulation between blood, tissue, lymphatics, and lymphoid organs. In comparison to lymphocyte migration directly from blood to lymph nodes (LN) or to non-lymphoid tissues, lymphocyte migration from tissues to LN via afferent lymphatics is less well understood. DC migration from peripheral tissues into lymphatics has received the most attention^1^ and depends on CCL21 gradients to terminal lymphatics using CCR7^2^. DC also migrate toward S1P^3^ and CXCL12 in to lymphatics^4^. Human DC require CD31 and CD99 in order to migrate across lymphatic endothelium^5^. The adhesion molecules ICAM-1, VCAM-1, E-selectin, and their corresponding ligands have all been implicated in DC migration across lymphatic endothelium^6^, and this interaction can influence DC function as well as migration^7^.

Like DC, T cells have been reported to use CCR7 to exit tissue and access lymphatics^8^. However, several reports suggested that CCR7 dependence is not uniformly required by T cells, as central memory CD4+ T cells do not require CCR7 to exit tissue, enter lymph, and infiltrate LN, while CD8+ central memory T cells do^9^. T cell migration from peripheral tissues to LNs via lymphatics can also be inhibited by treating T cells with sphingosine 1-phosphate (S1P) and S1P receptor 1 (S1PR1) agonists or by inflammation-induced increases in tissue S1P levels^10^. Regulation of T cell egress from tissues is important, as egress of CD8 and CD4 T cells has been shown to affect pathogen clearance and tissue damage^11^. Together, these findings underscore the complexity of the factors that regulate T cell tissue to lymphatic migration and the physiological importance of this process.

Others have found that neutrophil transmigration across lymphatic endothelium depends upon adhesion to the same ligands as T cells (ICAM-1, VCAM-1, and endothelial E-selectin), combined with CXCL8-dependent chemotaxis^12^. Common lymphatic endothelial and vascular endothelial receptor-1 (CLEVER-1) has been reported to be involved in the transmigration of monocytes, granulocytes, B cells, and T cells across lymphatic or lymphatic-like endothelium^13^.

Lymphatic TEM is also involved in leukocyte egress from LNs, as cells must pass through lymphatic endothelium before entering lymphatic sinuses and efferent lymphatic vessels. One important regulator of this process is S1P and its receptor S1PR1, present on multiple cell types including endothelial cells, cancer cells and T cells^14^. There is evidence that the S1P/S1PR1 axis acts both on T cells directly, with S1P serving as a signal for the T cell to leave the LN^15^, as well acting on endothelial cells to alter their barrier function^16^. The integrin LFA-1, chemokine receptor CCR7, and β2 adrenergic receptors have also been implicated in controlling lymphocyte egress from LNs^17^. However, as for migration into afferent lymphatics, the details of efferent migration remain incompletely described.

There are several *in vivo* models for lymphatic TEM, which include visualization of injected or endogenous cells interacting with the dense network of lymphatics in diverse anatomic locations^10, 12, 18, 19^. Several *in vitro* models of migration across lymphatic endothelial monolayers have been described but remain incompletely validated for LEC type, leukocyte subset, chemoattractant variables, directionality, or lymphatic variables. Johnson *et al*. reported a model for DC migration across primary human dermal LEC (HDLEC)^6^ in which DCs migrated from the basal to the apical (luminal) side, and relied on the production of chemoattractants by the HDLEC. This assay was also used to examine neutrophil migration across inflamed HDLEC layers^12^, and macrophage migration across LEC^20^. Others have described assays of cell migration across human LEC but in the luminal to basal direction, which may be less common *in vivo* ^21^. Swartz’s group refined an *in vitro* model by including modulated fluid flow through the lymphatic endothelial layer as well as across the luminal side of the layer^22^. Overall the model systems available have not explicitly tested whether migration is vectorial, not characterized whether the cells or cell lines reliably mirrored LEC phenotype and function^23^, or required extraordinary technical facilities^22^.

We present a simple system for lymphatic TEM with fluid flow through both lymphatic endothelial-like cell line (SVEC4-10) and primary LEC, validate vectorial lymphatic migration, show that human leukocytes and LEC have virtually identical properties, and demonstrate the ability to evaluate multiple cell types and chemoattractants. Importantly, this model has been validated *in vivo* ^10, 19^.

## Results

### Characterization of LEC phenotype

**Figure 1:**
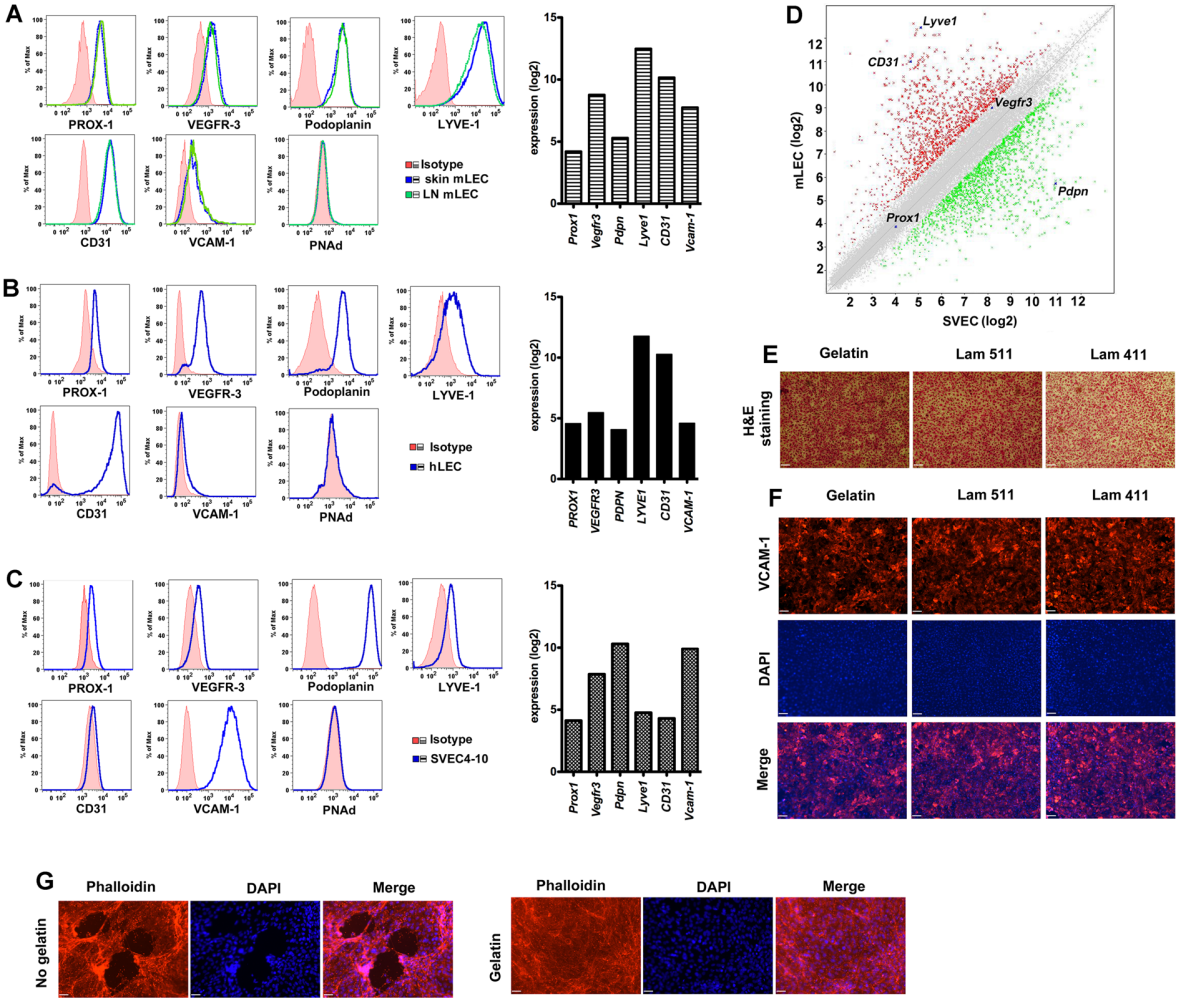
Characterization of LEC with flow cytometry and gene array. (**A**) Mouse skin or lymph node primary mLEC, (**B**) human skin primary hLEC, or (**C**) SVEC4-10 stained for the indicated markers and Affymetrix gene array. (**D**) Affymetrix gene array analyzed with GeneChip® Command Console® Software (AGCC) and Transcriptome Analysis Console (TAC) for SVEC4-10 vs mouse LEC. (**E**) H&E staining of SVEC4-10 layers grown on inserts coated with gelatin, laminin 411 and laminin 511, x10, scale bar 80 μm. (**F**) Fluorescence staining of SVEC4-10 layers for VCAM-1 with inserts coated with gelatin, laminin α4β1γ1 and laminin α5β1γ1, x10, scale bar 80 μm. (**G**) Fluorescence staining of phalloidin and DAPI for SVEC with inserts coated with/without gelatin. Representative of three independent experiments, triplicates for gene array, x10, scale bar 80 μm *P < 0.05.

We established the model using mouse and human primary LEC. LEC phenotype was confirmed for C57BL/6 mouse primary LEC (mLEC), and human skin primary LEC (hLEC) (Fig. 1). Murine LEC (Fig. 1A) expressed high amounts of the LEC-specifying transcription factor Prospero-type homeobox (PROX)-1^24^, VEGFR-3^25^; podoplanin/Gp38^26^; lymphatic vessel endothelial hyaluronan receptor (LYVE)-1^24^, and CD31; moderate VCAM-1; and no peripheral LN addressin (PNAd). Human LEC also expressed high amounts of PROX-1, VEGFR-3, podoplanin/Gp36, LYVE-1 and CD31; minimal VCAM-1; and no PNAd^6^. Gene expression analysis showed that human and mouse LEC were similar, to each other for expression of various homologous genes assessed via microarray including *PROX1* and *Prox1*, *VEGFR3* and *Vegfr3*, *PDPN* and *Pdpn*, *LYVE1* and *Lyve1*, *PECAM1* and *Pecam1*, and *VCAM-1 and Vcam-1* (Fig. 1A,B).

The C3H/HeJ mouse endothelial line SVEC4-10^10, 27^ was shown to have high amounts of the lymphatic-specific markers PROX-1, Podoplanin/Gp38, VEGFR-3, LYVE-1, CD31 and VCAM-1; and no PNAd (Fig. 1C). *Lyve1* and *Pecam1/CD31* were lower than primary LEC; *Pdpn/Gp38* and *Vcam1* higher; and *Vegfr3* and *Prox1* equivalent (Fig. 1C,D). These differences are commensurate with known *in vivo* variability in expression^6, 28^. *Prox1* and *Vegfr3* were high in SVEC4-10, and equivalent to expression in primary LEC, supporting its lymphatic character, since these genes specifically maintain lymphatic lineage and function^25, 28, 29^. To determine the significance of the gene expression differences noted above, we compared the gene array data from mLEC and SVEC4-10 by moderate Bayes t-test. P-values were corrected using a false discovery rate (FDR) = 0.05 to obtain adjusted p values. Out of 41345 probe sets, only 14 had adjusted p-values < 0.05. With unadjusted comparisons, 3317 probesets had p-values < 0.05; however, most of them (2067) were predicted to be by chance only (Supplementary Table 1). Of the 14 probe sets, 12 have known gene targets, and none of those genes has a role in specifying or maintaining the LEC lineage (Supplementary Table 2). These results indicate that SVEC4-10 are of lymphatic origin.

Afferent lymphatic endothelial cells are in contact with many stromal elements such as collagens and laminins. Others have shown effects of extracellular matrix stromal elements on LEC growth and morphology^30^. Therefore, to simulate *in vivo* conditions and study T cell migration via an *in vitro* transwell assay, LECs were seeded on transwell inserts coated with gelatin or the stromal components laminin α4β1γ1 and laminin α5β1γ1. The cells reached confluence after 2–3 days, verified by H&E staining and 3-D confocal microscopy (Fig. 1E, Supplementary Figure 1A), had similar growth characteristics and morphology, and expressed almost identical amounts of VCAM-1, ICAM-1and VE-cadherin on inserts coated with each stromal protein (Fig. 1F and data not shown). Imaging of the upper and lower surfaces of the inserts showed that the LEC and SVEC remained on one side of the insert membrane and extended lamellopodia-like processes across the membrane pores (Supplementary Figure 1A-B). Thus, the cell layers were confluent on one side of the membrane but were not confluent bilayers on both sides, and consistently DAPI were stained on the confluent side, not on another lamellopodia extension side (Supplementary Figure 1A-B). Without protein coating, the cells did not attach or reach 100% confluence (Fig. 1G). Additional imaging for the matrix proteins collagen and laminin showed that the endothelial cells produced stromal elements that likely further contributed to growth, stability, and adhesion (Supplementary 1C).

### T cells migrate through murine LECs in a vectorial fashion

LEC were seeded on either the upper (SVEC4-10/LEC) or lower (inverted or iSVEC4-10/iLEC) surface of the transwell insert to allow two separate configurations (Supplementary Figure 2). Leukocytes placed in the top chamber came into initial contact either with the apical (luminal) surface of SVEC4-10/LEC or the basal (abluminal) surface of iSVEC4-10/iLEC.

**Figure 2:**
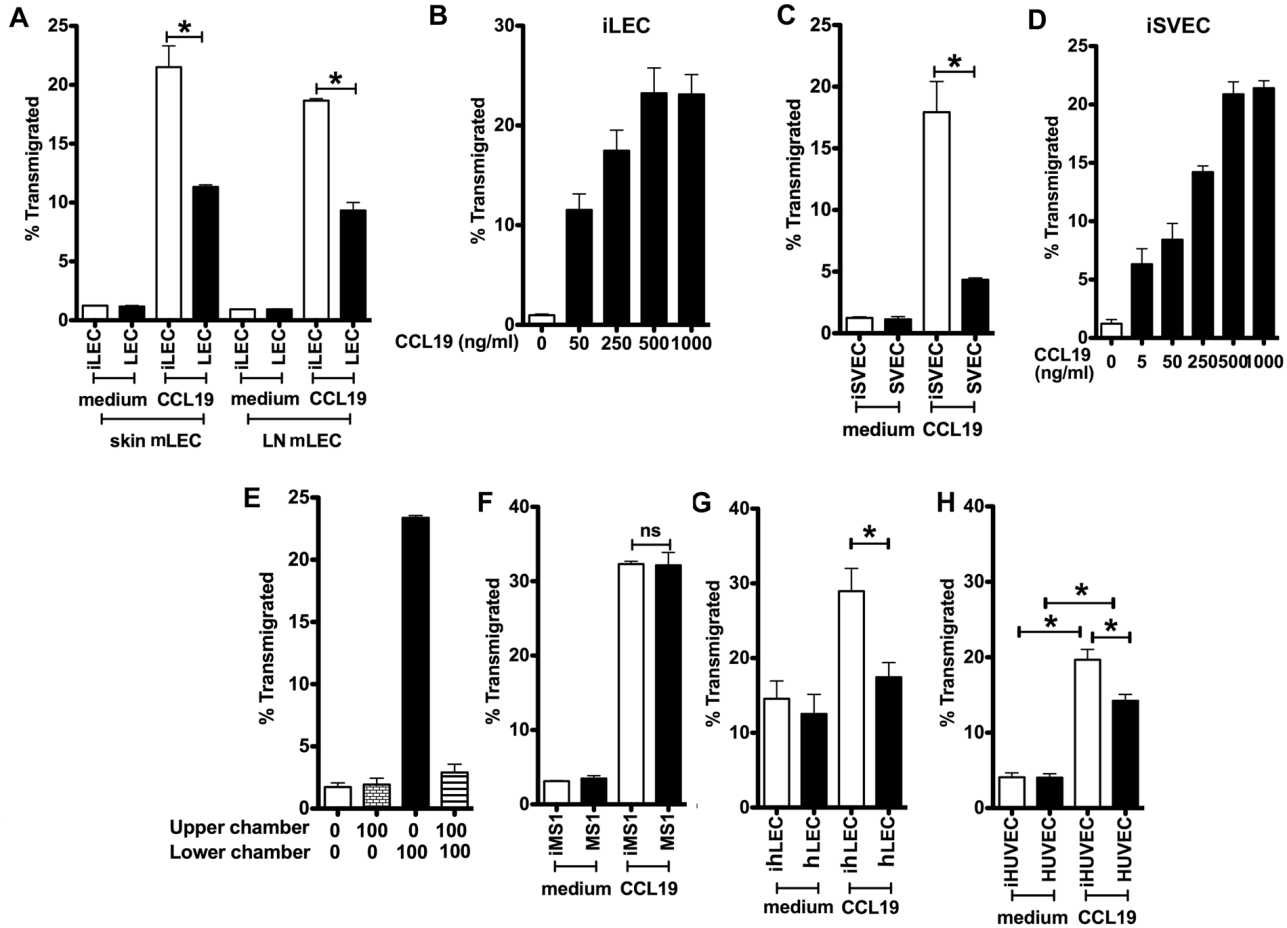
CD4 T cell migration across LEC or BEC. CD4 T cell migration across (**A**) skin or LN primary mLEC toward CCL19, (**B**) across skin iLEC toward CCL19 in dose response, and (**C**) across SVEC4-10 or iSVEC4-10 toward CCL19. (**D**) CD4 T cell migration across iSVEC4-10 toward various doses of CCL19. (**E**) CD4 T cell migration with CCL19 on upper, lower or both chambers across iSVEC. (**F**) CD4 T cell migration across iMS-1 or MS-1 toward CCL19. (**G**,**H**) Human CD4 T effector migration across (**G**) human primary hLEC or ihLEC layers toward CCL19, (**H**) Human CD4 T effector migration across HUVEC and ihLEC toward CCL19. Three independent experiments performed, all transwell assay has triplicates/experiments, representative of three independent experiments, all experiments pool together for human effector T cells *P < 0.05.

CD4 T cells migrated well toward CCL19 across mouse iLEC in the basal to apical direction, but poorly in the reverse direction (Fig. 2A). Migration was dose-dependent (Fig. 2B). CD4 T cells also migrated in a preferential vectorial fashion across iSVEC4-10 (Fig. 2C,D). CD4 T cell migration across iSVEC toward CCL19 was chemotactic but not chemokinetic or chemorepulsive (Fig. 2E). A C57BL/6 mouse blood vascular endothelial line^10^ did not support preferential migration (Fig. 2F), showing that CD4 T cells preferentially migrated across LEC, but not BEC, in a single direction but not bidirectionally. The vectorial migration was not due to permeability differences in the cell layers, as Evans blue dye dispersal as a measure of transcellular permeability was similar across iSVEC, iMS-1 and MS-1, and dispersed even more rapidly across SVEC (Supplementary Figure 3).

Human T cells also migrated in a preferential vectorial fashion across hLEC but not across the hBEC HUVECs. (Fig. 2G,H). This confirmed a preference for unidirectional human T cell migration across hLEC but not hBEC, similar to murine cells.

### Preferential vectorial migration occurs for multiple chemotactic signals

**Figure 3:**
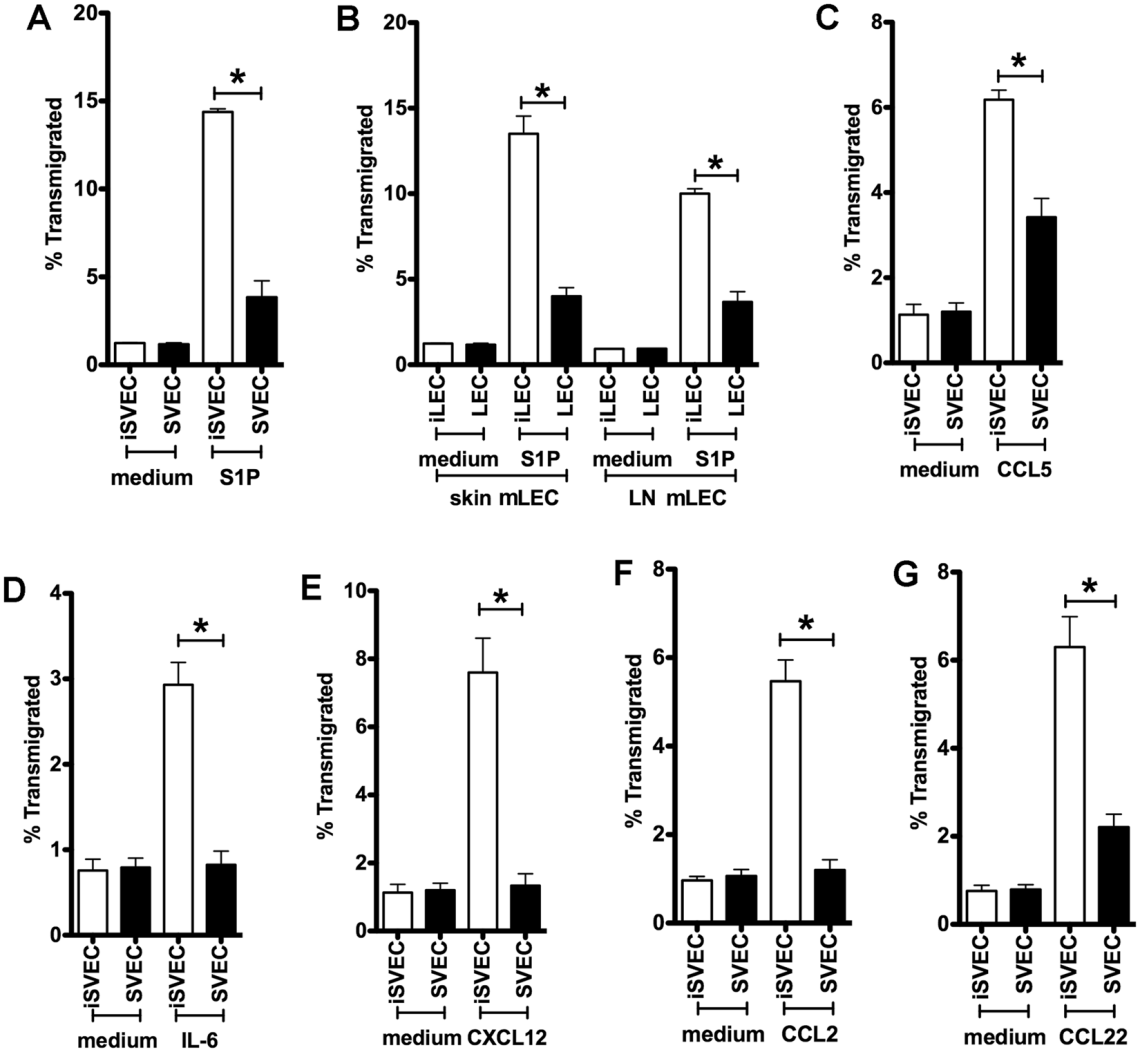
CD4 T cell migration toward various chemokines or cytokines. CD4 T cell migration across iSVEC4-10 or SVEC4-10 toward (**A**) S1P, (**C**) CCL5, (**D**) IL-6, (**E**) CXCL12, (**F**) CCL2, and (**G**) CCL22, or (**B**) across mLEC toward S1P. Representative of three independent experiments with triplicate wells in each assay *P < 0.05.

Although CCR7 is critical for CD4 T cell afferent lymphatic migration, many other chemokines also play important roles in CD4 T cell homing into and positioning within LN^31^. Additional homeostatic and inflammatory chemokines and cytokines were tested at published optimal concentrations^10, 19, 32^, and each induced CD4 T cell unidirectional migration across iSVEC4-10 or imLEC, including S1P (Fig. 3A,B), CCL5 (Fig. 3C), IL-6 (Fig. 3D), CXCL12 (Fig. 3E), CCL2 (Fig. 3F) and CCL22 (Fig. 3G). CCL19 and CCL21 generally induced much greater levels of migration compared to the other ligands, commensurate with observations regarding the role of CCR7 in lymphatic migration^8^. These results demonstrate that many chemoattractants are active in this assay, and the preferential vectorial migration is a general characteristic of the model, not dependent on only a single receptor or ligand.

### Stromal fibers, lymphatic flow, and chemokine distribution influence the LEC microenvironment

**Figure 4:**
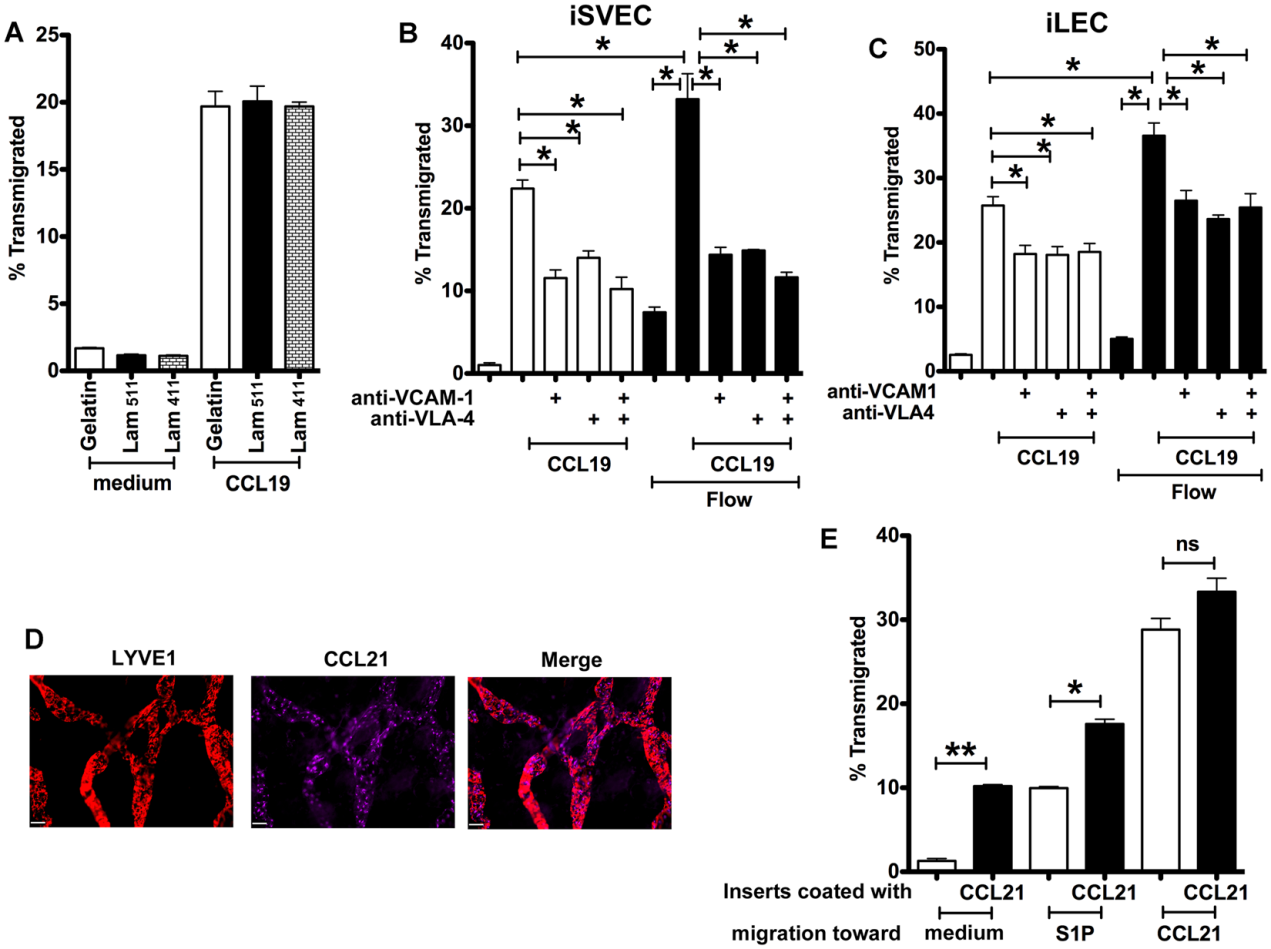
Stromal fibers, fluid flow, and chemokine position regulate LEC and migration. (**A**) CD4 T cell migration toward CCL19 across iSVEC4-10 with inserts coated with gelatin, laminin α4β1γ1 and laminin α5β1γ1. (B-C) CD4 T cell migration across iSVEC4-10 (**B**) or iLEC (**C**) toward CCL19 with or without flow, and blocked with anti-VLA-4 and anti-VCAM-1 mAb. (**D**) Ear whole mount staining of CCL21 and Lyve-1, x20, scale bar 42 μm. (**E**) CD4 T cell migration toward S1P or CCL21 across iSVEC4-10 coated with CCL21. Representative of three independent experiments with triplicate wells in each assay *P < 0.05.

We tested whether inserts pre-coated with the stromal components gelatin, laminin α4β1γ1 and laminin α5β1γ1 affected migration. CD4 T cells migrated equally toward CCL19, independent of which proteins were used to coat the insert (Fig. 4A). Thus, while stromal components were important for LEC growth and establishment of a monolayer, gelatin and two different isoforms of laminins seem to function similarly in this regard.

Translymphatic fluid flow affects the phenotype and function of lymphatic monolayers, as occurs *in vivo* ^33^. To mimic interstitial-type fluid flow, we altered the standard migration assay in which 600 µl is placed in the lower well and 100 µl in the upper well, creating a single static fluid column. Instead, the lower well was filled with the minimum volume to cover the bottom of the insert (360 µl), while the upper well was maximally filled (340 µl). This resulted in a pressure differential of 0.8–0.9 cm H_2_O, similar to interstitial pressure *in vivo* ^34^. During the 3 h assay, approximately 47.5 μl of medium flowed from the upper to the lower chamber, equivalent to an average flow rate of 15.8 ul/h. This fluid flow enhanced CD4 T cell migration across iSVEC4-10 (Fig. 4B) or iLEC (Fig. 4C) toward CCL19.

CD4 T cell TEM migration is dependent on both VLA-4-VCAM-1 dependent and independent interactions^10, 35^. TEM could be inhibited under both flow and no flow conditions with mAbs to VLA-4 and VCAM-1 (Fig. 4B,C). The additional migration observed under flow conditions was all inhibited by these mAbs, showing that increased migration was not simply due to passive carriage of the T cells through the LEC, but depended on specific receptor-ligand interactions between the T cells and the LEC. The results also confirmed that there were was significant residual migration in the presence of blocking mAbs, revealing VLA-4/VCAM-1 independent components to TEM, as previously noted^10, 18, 19, 41^.

While the system described so far uses soluble chemoattractants to assay migration across lymphatic endothelium, chemokines and other chemoattractants are not always in the soluble phase *in vivo*. In particular CCL21 immobilized on lymphatic endothelium in discrete puncta is required for DC migration^2, 36^. Whole mount staining of ear pinnae lymphatics showed that CCL21 is distributed in patches dispersed along the afferent lymphatics^36, 37^ (Fig. 4D). To mimic this *in vivo* distribution, 500 ng/ml CCL21 in 100 μl gelatin was coated, incubated for one hour at 37 °C, and then excess chemokine washed from the basal surfaces of iSVEC4-10 immediately before the migration assay and compared to the conventional addition of a much larger quantity of CCL21 (500 ng/ml CCL21 in 600 μl medium) to the bottom chamber. CD4 T cells migrated effectively to the CCL21 coating (Fig. 4E). Therefore, the model was able to mimic the distribution of chemokine fixed along the LEC surface and did not necessarily require an artificial chemokine gradient created by adding a large amount of soluble chemokine to the lower chamber.

### Inflammation regulates LEC phenotype and CD4 T cell migration

**Figure 5:**
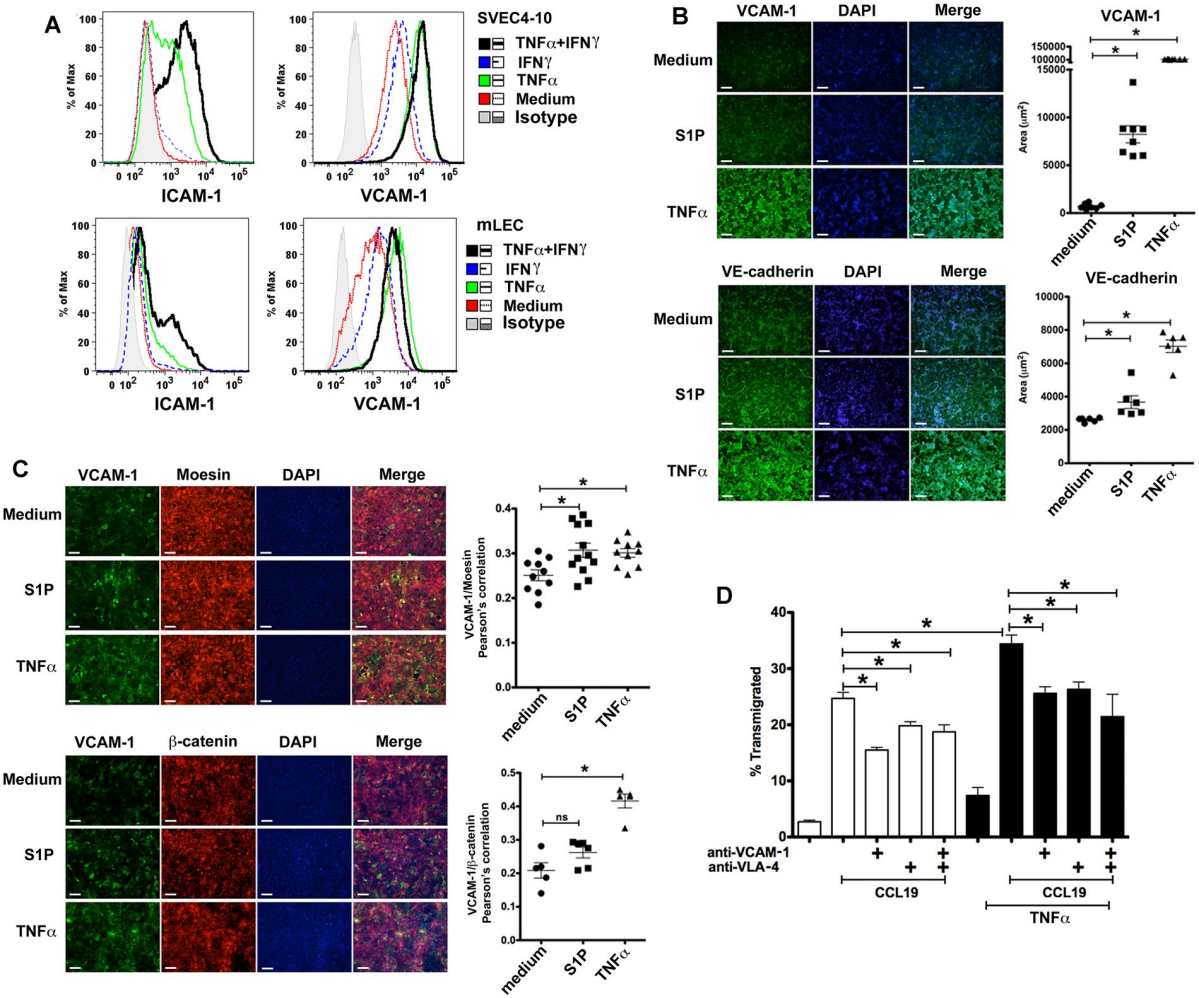
Adhesion molecule expression and CD4 T cell migration across SVEC4-10 layers treated with TNFα, IFNγ, or S1P. (**A**) SVEC4-10 or mLEC treated with TNFα and/or IFNγ, stained for VCAM-1 and ICAM-1, and analyzed with flow cytometry. (**B**) Fluorescent staining of SVEC4-10 treated with medium, TNFα or S1P for VCAM-1 and VE-cadherin (left) and quantitation of area labeled (right), x20, scale bar 42 μm. (**C**) Fluorescent staining of SVEC4-10 layers treated with medium, TNFα or S1P for VCAM-1, moesin and β-catenin (left) and quantitation of label clustering (right), x20, scale bar 42 μm. (**D**) CD4 T cell migration across iSVEC4-10 treated with TNFα, and blocked with anti-VLA-4 and/or anti-VCAM-1 mAbs. Representative of three independent experiments with triplicate wells in each assay *P < 0.05.

The interactions between murine CD4 T cell surface integrins and LEC VCAM-1 and ICAM-1 mediated CD4 T cell TEM^35^ (Fig. 4B,C). Low levels of VCAM-1 and ICAM-1 were expressed by murine LEC under basal conditions (Fig. 1), and were up-regulated by the pro-inflammatory cytokines TNFα or IFNγ^6, 10^. Confirming this response, both mLEC and SVEC4-10 up-regulated VCAM-1 and ICAM-1 expression in response to TNFα and IFNγ treatment (Fig. 5A). Immunofluorescent staining of SVEC4-10 and mLEC layers showed TNFα increased VCAM-1, ICAM-1 and VE-cadherin expression (Fig. 5B, Supplementary Figures 4 and 5). S1P is not only an attractant for leukocytes, but also regulates endothelial cell integrity^14^. S1P treatment increased VCAM-1 and VE-cadherin but not ICAM-1 expression (Fig. 5B, Supplementary Figures 4 and 5).

VLA-4-VCAM-1 interactions recruit more VCAM-1, actin, moesin and β-catenin to the site of adhesion to promote leukocyte TEM^38^. TNFα and S1P enhanced the co-localization of VCAM-1 and moesin, but not VCAM-1 and F-actin (phalloidin) for SVEC layers (Fig. 5C, Supplementary Figures 6 and 7). TNFα also enhanced VCAM-1 co-localization with β-catenin, and there was a non-significant trend for S1P to increase co-localization for SVEC layers (Fig. 5C and Supplementary Figure 7). Thus, the T cell-LEC interactions are consistent with canonical integrin function and signaling.

TNFα treatment of LEC increased CD4 T cell migration (Fig. 5D), which was VLA-4-VCAM-1 dependent (Fig. 5D). The additional migration observed under inflammatory conditions was only partly inhibited by these mAbs, showing that increased migration may be due to other mechanisms governing T cell and LEC interactions. The results again demonstrated that there were both VLA-4/VCAM-1 dependent and independent components to CD4 T cell TEM.

Endogenous chemokines may be differentially expressed by apical and basolateral surfaces of LEC^12^. Therefore, we collected conditioned medium from the upper and lower chambers of inserts with LEC treated or untreated with TNFα. The media were then used to drive chemotaxis and/or chemokinesis by placing them in the lower or upper chambers, respectively, of a second set of transwells. The results showed that conditioned medium had minimal effects on CD4 T cell TEM (Supplementary Figure 8). Overall, TNFα treatment enhanced CD4 T cell migration by regulation of adhesion molecule expression and function, but not by enhancing the expression of secreted endogenous chemotactic molecules.

### Other leukocyte subsets and cancer cells display vectorial migration

**Figure 6:**
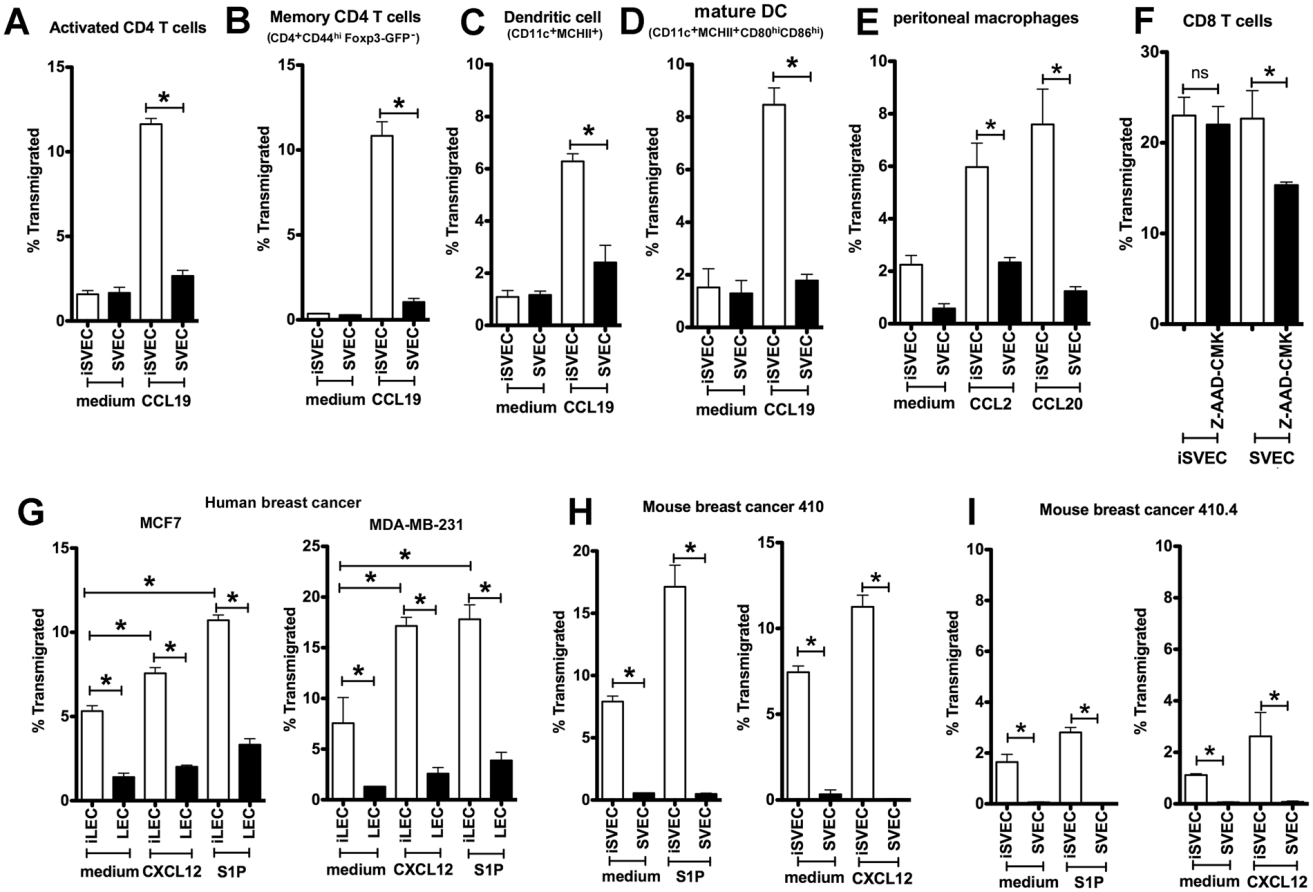
Diverse cell types migrate in a chemokine-driven vectorial fashion across LEC. (**A**–**D**) Activated CD4 T cells (**A**), memory CD4 T cells (CD4^+^CD44^hi^Foxp3-GFP^−^) (**B**), DC (CD11c^+^MHCII^+^) (**C**), and mature DC (CD11c^+^MHCII^+^CD86^hi^CD80^hi^) (**D**) migration across iSVEC4-10 or SVEC4-10 toward CCL19. (**E**) Peritoneal macrophages migration toward CCL2 and CCL20 across iSVEC4-10 or SVEC4-10. (**F**) CD8 T cells migration across iSVEC4-10 or SVEC4-10 toward CCL19. (**G**) Human breast carcinoma lines MCF7 (left) and MDA-MB-231 (right), migration across hLEC and ihLEC toward S1P and CXCL12. (**H**,**I**) Mouse breast cancer cell lines 410 (**H**) and 410.4 (**I**) migration across SVEC4-10, iSVEC4-10, toward S1P or CXCL12. Representative of three independent experiments with triplicate wells in each assay *P < 0.05.

To test whether the directional migration was a generalized phenomenon, a variety of other leukocyte subsets were evaluated. Activated CD4 T cells (Fig. 6A), memory CD4 T cells (Fig. 6B), DC (Fig. 6C), mature DC (Fig. 6D) and macrophages (Fig. 6E) all migrated far more efficiently across iSVEC4-10 than SVEC4-10 toward CCL19. In contrast, naïve CD8 T cells migrated equally across iSVEC4-10 and SVEC4-10 (Fig. 6F). One possible explanation for the different migration behavior of CD8 T cells is their high expression of granzymes compared to CD4 T cells, particularly in light of reports that granzymes regulate CD8 T cell transmigration through endothelia^39^. To test this, CD8 T cells were pretreated with the granzyme B inhibitor Z-AAD-CMK before migration through SVEC4-10 and iSVEC4-10 layers. The results showed that Z-AAD-CMK partly inhibited CD8 T cell transmigration across SVEC4-10 but not iSVEC4-10 (Fig. 6F). This result suggests that the ability of CD8 T cells to migrate across SVEC4-10 may be because of their elevated expression of active granzyme B.

Since carcinoma cell trafficking into LN requires cell migration and chemotaxis^40^, the human breast cancer cell lines MCF7 and MDA-MB-231 were evaluated. These human carcinomas migrated more across ihLEC than hLEC to either S1P or CXCL12 (Fig. 6G). Similarly, mouse breast cancer cell lines 410 and 410.4 migrated more across iSVEC4-10 than SVEC4-10 toward S1P or CXCL12 (Fig. 6H–I). Thus, the observation of preferential vectorial migration through human and murine LECs is true not only for diverse leukocyte subsets, but also for carcinoma cell lines.

## Discussion

Here we described a simple, flexible, affordable system for evaluating cell migration across lymphatic endothelium *in vitro*. One can select the endothelial cell type, migrating cell type, extra-cellular matrix components, fluid flow, chemoattractant, and physical partitioning or distribution of the chemoattractant (soluble or adsorbed). Removal of the inserts during or after the migration assay allows fixed and live time-lapse imaging^10^. SVEC4-10 was validated to behave almost identically to primary LEC. The addition of fluid flow/pressure differential posed no further technical or cost barrier. SVEC4-10, mouse primary LEC, and human primary LEC all permitted preferential vectorial migration; in contrast primary BEC and BEC lines did not display such properties. Unidirectional migration across LEC recapitulates the *in vivo* preference for basal (abluminal) to apical (luminal) directionality as occurs during cell movement from non-lymphoid tissues into afferent lymphatics, and during movement into cortical or medullary sinuses prior to egress from lymph nodes.

There are several *in vivo* models for examining trans-lymphatic endothelial migration and all have challenges and limitations. We previously reported using visualization of injected or endogenous cells interacting with the dense network of lymphatics in the ear pinna using whole mount ear preparations^10^. However, this only gives a static snapshot at one point in time of migrating cell interactions with lymphatics. Others have reported tracking cells injected into this and other sites^10, 12, 18, 19^. We have also examined movement of injected T cells in live ear explants^41^. These assays often require large numbers of injected cells for efficient detection in the draining lymph nodes, and entail the variability, complexity, and cost of using live animals. Our *in vitro* system required only as many variables as the investigator chose to introduce, including the migrating cell type, extra-cellular matrix components, fluid flow, and chemo-attractant type and position. This system could be used for initial discovery, then to sequentially build complexity before validating select findings *in vivo*. While acknowledging that this *in vitro* two dimensional system lacks the full complexity of the *in vivo* 3 dimension environment, this model has already been validated by our own results *in vivo* using multiple different genetic ablations, blocking antibodies, and small molecule inhibitors^10, 19^.

We validated that SVEC4-10 is an LEC line via several assays. First, the protein markers expressed by SVEC4-10, including the CD31 and Podoplanin/Gp38 surface markers often used to identify LEC^42^, as well as the lymphatic lineage specifying PROX-1^43^, were broadly equivalent to the pattern of expression of those markers by primary mouse LEC. Second, cells migrated across primary mouse LEC and SVEC4-10 layers to a similar degree. Importantly, both only supported robust transmigration of most leukocyte subsets and carcinomas across them in the basal to apical direction, the one exception being CD8 T cells which do not show a directional preference for either cell. Finally, via microarray analysis of gene expression profiles SVEC4-10 and primary mouse skin LEC were again broadly similar. One major advantage of the present model lies in the definition, use, and validation of the immortalized SVEC4-10 LEC line. While primary cells are often a better choice for accurately modeling biological events, they may be altered by the process of isolation from their normal tissues of residence, tend to de-differentiate *in vitro* ^23^, have a limited life span, be available in only limited quantities, be expensive to manufacture, and often be difficult to transfect or transduce. In contrast, cells lines, while they may deviate from primary cells in some phenotypic or functional ways, tend to be more stable over time, grow indefinitely, and are typically more amenable to genetic manipulation. The similarity between early passage primary LEC and SVEC4-10 cells in our functional assays and by microarray gene expression analysis supports the use of SVEC4-10 in place of primary LEC for screening and discovery assays.

The reason for the outlier behavior of CD8 T cell non-vectorial migration across LEC is also not clear. There is evidence that memory CD4 and CD8 T cells have differing propensities to migrate from tissues into LNs with CD4 migrating more than CD8 T cells^9, 44^. However, the molecular determinants of these differences remain undetermined. On the other hand, the dynamics of CD4 and CD8 T cell egress from LNs during infection appear to be similar^45^. Others have found that CD4 and CD8 T cells both migrated more efficiently across blood endothelium in the luminal to basal direction than in the reverse direction, but that migration of both T cell subsets was similar in both directions^46^. It is possible that the behavior we observed in CD8 T cells represents an ability to leave lymphatic vessels or move from lymphatic sinuses in LNs back into the LN parenchyma. Indeed, T cells engaging in the latter behavior have been described in an elegant live imaging study^15^, but the T cells were not identified specifically as either CD4 or CD8 T cells. It is also important to note that our current data with CD8 T cell migration are *in vitro* and not yet confirmed *in vivo*. Previous studies showed that CD8 cells employed granzymes to traverse BEC^39^, and BEC have a true basement membrane, while LEC do not. Nonetheless, there is surrounding stroma and LEC junctions which could be susceptible to proteolytic cleavage. Immunohistochemical staining of iSVEC layers showed the extracellular matrix components collagen and laminin (Supplementary Figure 1D), which could be potential targets of granzyme B^47^. It is not yet clear if our *in vitro* model most resembles initial lymphatics where there are many gaps between cells, or collecting lymphatics where there are well defined intercellular junctions and surrounding stoma. Thus, cells may employ different mechanisms when traversing different lymphatic beds. All these considerations underscore the complexity of cell interactions with lymphatic endothelium and the extent of the unknowns remaining in this area.

LEC and BEC differed in the directionality of cell migration possible across them. With the exception of CD8 T cells that migrated equivalently in both directions, all cells tested migrated much more across LEC in the basal to apical than in the reverse direction, in which migration was very poor. In contrast all cells tested migrated across the BEC line MS-1 cells equivalently in both directions. It is possible that LECs prevent migration in the apical to basal direction where as BEC support migration in both directions. Another possibility is that this difference may relate to specialization for disparate environments and functions. BEC line the lumens of blood vessels, and the luminal surface is exposed to blood flow and shear forces under relatively higher and constant flow parameters. In contrast, LEC are exposed to discontinuous, lower pressures, and lower rates of transmural rather than shear fluid flow. Thus, the *in vitro* assay may better recapitulate the *in vivo* situation for LEC than for BEC.

Another structural difference is that in the post-capillary venules where blood to tissue extravasation occurs, BEC are lined on their basal surfaces with a dense network of basement membrane and pericytes^48^. In contrast, a single LEC layer with minimal or no basement membrane comprises the wall of lymphatic vessels^48^. Thus, the basal environments of BEC and LEC differ dramatically, altering the way leukocytes can interact with them. Possibly, the opportunities for leukocytes to interact with the basal surface of BEC *in vivo* may be limited. The notion that initial leukocyte interaction with the basal surface of BECs is not common is supported by the relative rarity of so-called “reverse transmigration” from tissue directly into blood vessels in most species, with only certain subsets of monocytes^49^ and neutrophils^50^ convincingly identified as using this route in non-lymphoid tissues.

Our model does not differentiate between migration into afferent lymphatic vessels in non-lymphoid tissues versus migration across lymphatic barriers in the subcapsular, cortical, or medullary sinuses in the LN. While, the supplier from which we obtained primary LEC has both skin LEC and LN LEC available, we did not observe phenotypic or functional differences between the two. The lack of differences may relate to observations that LEC rapidly dedifferentiate *in vitro* ^23^, likely due to lack of normal surrounding structures and soluble signals. It is also important to note that LEC in culture have junctional adhesion molecules surrounding the cell and do not replicate the cellular discontinuities and button like structures observed *in vivo* in initial afferent lymphatic capillaries^55^. It may be possible to tailor *in vitro* assays to mimic entry into lymphatic vessels in the periphery or movement across lymphatic endothelial barrier in the LN by choosing the relevant leukocyte subset and LEC source and altering the chemoattractants and extra-cellular matrix components. Results from other work in our lab showed that Treg egress from non-lymphoid tissues depended upon expression of lymphotoxin, while egress from lymph nodes did not^41^. Treg transmigration across murine skin LEC and SVEC4-10 also depended upon lymphotoxin, suggesting that the *in vitro* assay may more faithfully replicate afferent than efferent lymphatic TEM.

While most study of lymphatic TEM has focused on leukocytes, our assay can analyze other cells. Tumor invasion of lymphatics is an important step in metastasis^51^. The MCF-7 human breast cancer line can induce gaps to form in lymphatic monolayers *in vitro*, but transmigration across those monolayers was not measured^52^. Conditioned medium from primary human LEC induced migration of the human breast cancer lines MDA-MB-435, MCF-7 and MDA-MB-231, and the melanoma line SK-MEL-25 *in vitro*, but migration of tumor across LEC was not determined, although the tumor cells did transmigrate across the floor of the subcapsular sinus^40^. Giampieri and colleagues observed tumor cells migrating into lymphatic vessels using intra-vital time lapse imaging^53^, but the amount of experimental manipulation possible in such a system is less than in ours, and the technical barriers to performing such studies are high. Swartz’s group developed an extremely impressive model to examine lymphatic flow and cell migration using human cancer lines and human primary LEC^22^, but entails a setup cost and expertise that is likely a barrier to most research groups. The model presented here could be exploited to study the molecular determinants of tumor cell lymphatic TEM. We demonstrated that mouse and human breast carcinoma migration across LEC could be easily measured, responsive to different chemotactic signals (CXCL12, S1P), and display preference for the basal to apical direction as the leukocyte subsets. This finding appears to be novel, though its molecular underpinnings remain to be determined. S1P has been described to be a prometastatic factor in human rhabdosarcoma^54^ and important for cancer cell migration and metastasis^55^. CXCL12 is known to be an important factor in breast carcinoma metastases and biology^56^. Our simplified model for transmural fluid flow could also be important for studying cancer cell migration as there is evidence that fluid flow in the tumor is dysregulated and influences cancer cell migration^57^. The system presented here represents an opportunity to explore tumor cell-LEC interactions, and result in important new insights about how this interaction regulates cancer metastasis via the lymphatics.

## Methods

All methods were performed in accordance with and approval by the University of Maryland School of Medicine for handling of radioactivity, cell lines, hazardous reagents, and potential pathogens.

### Mice

C57BL/6 mice were purchased from The Jackson Laboratory (Bar Harbor, ME). Foxp3GFP^58^ mice on a C57BL/6 background were from Dr. A. Rudensky (Memorial Sloan Kettering Cancer Center). All experiments involving mice were reviewed, approved, and performed in accordance by and with the Institutional Animal Care and Use Committee of the University of Maryland School of Medicine.

### Cell lines

MS-1 cells (CRL-2279) and SVEC4-10 cells (CRL-2181) were from the American Type Culture Collection (Manassas, VA). Human breast cancer cell lines MCF-7^59^ (low metastatic potential) and MDA-MB-231^60^ were from Jeffrey Winkles (University of Maryland). MS-1, SVEC4-10, MCF7 and MDA-MB-231 cells were cultured in DMEM containing 10% (vol/vol) FBS (Benchmark, Gemini, West Sacramento, CA), 2 mM L-glutamine (Lonza, Allendale, NJ), 100 IU/ml penicillin (Lonza), and 100 ug/ml streptomycin (Lonza). Mouse breast cancer cell lines 410 and 410.4 were from Amy Fulton (University of Maryland). 410 and 410.4 were cultured in RPMI-1640 10% FBS, 2 mM L-glutamine, 100 IU/ml penicillin), 100 μg/ml streptomycin, and 1× nonessential amino acids (Lonza). All cell lines were cultured in 5% CO_2_ atmosphere. Cell lines were maintained in 75 cm^2^ vented culture flasks (Corning) in a horizontal orientation until they reached 70–85% confluence, then treated with trypsin-EDTA (Gibco, New York) and passaged to new flasks at a dilution of 1:6. Cultures were used up to passage five for SVEC4-10, and 6 for all other primary cells and cell lines. MS-1, and SVEC4-10 were treated with 0.5% Trypsin EDTA for 5–7 minutes at 37 °C for detachment; MCF-7 and MDA-MB-231 were treated with 0.25% Trypsin EDTA for 5–7 minutes at 37 °C for detachment; 410, and 410.4 were treated with 0.25% Trypsin EDTA for 10–12 minutes at 37 °C for detachment; and 66.1 were treated with 0.05% Trypsin EDTA for 2–4 minutes at room temperature for detachment.

### Primary cells

C57BL/6 mouse skin primary LEC and human skin primary LEC were purchased from Cell Biologics (Chicago, IL), and cultured according to the manufacturer’s instructions (mouse endothelial cell medium supplemented with 5% FBS, 2 mM L-glutamine, 100 IU/ml penicillin, VEGF, ECGS, Heparin, EGF, hydrocortisone; human endothelial cell medium with 10% FBS, 2% endothelial cell supplement, 2 mM L-glutamine, 100 IU/ml penicillin, VEGF, Heparin, EGF, FGF, hydrocortisone). Human Teff were isolated and grown as previously described. Briefly, naive CD4+ T cells (CD4+25−127+CD45RA+) were sorted from nonmobilized peripheral blood apheresis products (Memorial Blood Center, St. Paul, MN)^61^. Naive CD4 T cells were stimulated with anti-CD3 mAb (Orthoclone OKT3, Janssen-Cilag, Raritan, NJ) and a K562 cell line irradiated with 10,000 cGray (KT) engineered to express CD86 and the high affinity Fc Receptor (CD64) at a T cell:KT ratio of 2:1 in the presence of IL-2 to produce “effectors”. Mouse CD4 T cells (naïve, activated, memory), CD8 T cells, and DC were prepared according to manufacturer’s protocols (STEMCELL Technologies, Inc., Vancouver, Canada). Naïve CD4 T cells were enriched by STEMCELL CD4 T cell negative selection kit, which were negatively selected with anti-CD8a, anti-CD11b, anti-CD11c, anti-CD19, anti-CD45R/B220, anti-CD49b, anti-TCRγ/δ and anti-TER119. Naïve CD8 T cells were negatively selected with anti-CD4, anti-CD11b, anti-CD11c, anti-CD19, anti-CD45R/B220, anti-CD49b, anti-TCRγ/δ and anti-TER119. Dendritic cells were selected from splenocytes with CD11c positive selection kits (STEMCELL Technologies, Inc., Vancouver, Canada). Activated CD4 T cells were stimulated with plated-bound anti-CD3 for 3days. Memory CD4 T cells were flow sorted from Foxp3-GFP mice by gating on CD4^+^CD44^hi^Fop3-GFP^−^CD25^−^. Peritoneal macrophages were from Dr. Stefanie Vogel’s lab (University of Maryland). In brief, 3 ml of 3% thioglycollate medium (Remel, Lenexa, KS, USA) was injected into the peritoneal cavity of each mouse, and macrophages were collected from the peritoneal washings 3 days later, seeded into 100mm petri-dish overnight, and cells adherent to the dishes were used for transwell assay.

### Reagents

S1P was from Avanti Polar Lipids (Alabaster, AL). Recombinant murine CCL19, CCL5, CCL22, CCL2, CXCL12, CXCL10, IL-6, TNFα and IFNγ were from R&D Systems (Minneapolis, MN). Anti-VCAM-1 (clone 429), anti-ICAM-1 (clone YN1/1), anti-CD4 (GK1.5), anti-CD25 (clone PC61.5), anti-CD44 (clone IM7) and anti-TNFα were from eBioscience (San Diego, CA). Anti-human CD31, anti-human LYVE1, anti-human VEGFR3, anti-human GP38, anti-human ICAM-1 and anti-human VCAM-1 antibodies were from Biolegend (San Diego, CA). Anti-VE-cadherin was from BD PharMingen (San Diego, CA). AlexFluor-555 phalloidin was from Life Technologies (Grand Island, NY). Anti-moesin was from Cellsignaling (Danvers, MA). Anti-β-catenin, anti-collagen and anti-laminin were from Abcam (Cambridge, UK). Anti-prox1 antibody was from Bioss (Woburn, MA). Recombinant human laminin 511 was from Biolamina AB (Sundbyberg, Sweden). EIA grade gelatin was from Bio-Rad (Berkely, CA).

### Immunofluorescent Staining

SVEC4-10 monolayers were stained with anti-VCAM-1, anti-ICAM-1 and anti-VE-cadherin for 1 h at 4 °C, then incubated with FITC labeled donkey anti-rat secondary antibody (Jackson ImmunoResearch, West Grove, PA) for 30 min at 4 °C. The inserts were washed with PBS, and fixed with 3% PFA at 4 °C for 10 minutes. The insert membranes were removed from transwell and mounted on slides. Inserts were permeabilized with 0.1% Triton-X-100 in PBS for 5 minutes at 4 °C, stained with anti-moesin or anti-β-catenin overnight at 4 °C, or Alex Flour 555 labeled phalloidin for 30 minutes at 4 °C, then incubated with secondary antibody for 1 hour at 4 °C. Quantitative analysis was performed with Volocity 3D Image Analysis Software to demonstrate the distribution of different molecules on cells, measure the density of different staining, and calculate the Pearson’s Correlation coefficients for molecule co-localization (PerkinElmer, Waltham, MA).

### Whole Mount Ear Staining

Ears were collected and peeled into two halves, fixed with 3% paraformaldehyde (PFA) in PBS for 5 minutes at 4 °C, permeabilized with 1% Triton X in PBS for 30 minutes at 4 °C, incubated with 5% donkey serum for 30 minutes at 4 °C, and incubated with the primary antibody at 4 °C overnight. The ears were then washed with PBS, incubated with secondary antibody for 4 hours at 4 °C, washed with PBS, and fixed with 3% PFA at 4 °C for 10 minutes. The stained ears were analyzed by fluorescence microscopy (Nikon Eclipse E800, Nikon Co., Tokyo, Japan).

### Flow cytometry

Staining was performed with the indicated antibody (Table 1) at 4 °C for 30 minutes according to the manufacturer’s instructions. Cells were analyzed with an LSRFortessa cell analyzer (BD Biosciences, San Diego, CA). Data were analyzed with FlowJo software v 8.8.7 (Tree Star, Ashland, OR).

**Table 1:** Antibodies for flow cytometry.

Antibody Specificity	Target Species	Clone	Fluorochrome	Vendor
CD31	Mouse	390	Allophycocyanin	eBioscience
CD4	Mouse	GK1.5	Allophycocyanin -eFluor 780	eBioscience
CD8	Mouse	53–6.7	Allophycocyanin	eBioscience
CD44	Mouse/Human	IM7	PE-Cy7	eBioscience
CD25	Mouse	PC61.5	PE	eBioscience
VCAM-1	Mouse	429	Unconjugated Or Allophycocyanin	eBioscience
ICAM-1	Mouse	YN1	Allophycocyanin	eBioscience
E-selectin (CD62E)	Mouse	10E9.6	PE	eBioscience
P-selectin (CD62P)	Mouse	RB40.34	PE	eBioscience
E-cadherin	Mouse	DECMA-1	PE	eBioscience
Podoplanin (GP38)	Mouse	eBio8.1.1	PE	eBioscience
VEGFR3	Mouse	m4F-31C1	Unconjugated	Dr. Pytowski (ImClone Systems, Eli Lilly and Company)
LYVE1	Human	537028	Allophycocyanin	R7D systems
CD31	Human	WM59	Pacific Blue	Biolegend
Podoplanin (GP38)	Human	NC-08	PE	Biolegend
VEGFR3	Human	9D9F9	Allophycocyanin	Biolegend
VCAM1	Human	STA	PE	Biolegend
ICAM1	Human	HCD54	Alexa Fluor488	Biolegend
Prox1	Mouse/human	Bs-2774	Alexa Fluor488	Bioss
VE-cadherin	Mouse	55-7H1	Unconjugated	BD
PNAd	Mouse/human	MECA-79	Unconjugated	Biolegend

### Gene expression assay

Total RNA from SVEC4-10 and mouse primary and human primary LEC was isolated with RNeasy Mini Kit (Qiagen, Valencia, CA), and residual DNA was removed with on-column DNase treatment (Qiagen). Highly purified RNA (A260/A280 ratio between 1.9–2.1, no degradation of RNA RIN (RNA integrity number) >7.0) were assayed with mouse and human Affymetrix gene ST2 arrays (Affymetrix, Santa Clara, CA). The data were acquired and analyzed with software according to the company’s instructions.

### Transendothelial growth and migration assays

Confluent MS-1, SVEC4-10, mouse primary LEC, human primary LEC and HUVEC layers grown in flasks were gently detached with trypsin, and 7.5 × 10^4^ cell lines (MS-1 and SVEC4-10) or 15 × 10^4^ primary cells (mLEC, hLEC and HUVEC) were seeded on the upper surface of a transwell insert coated with 0.2% (wt/vol) gelatin and cultured. After 3 days of culture, the integrity of the confluent layers was assessed with a quick hematoxylin and eosin stain (VWR, Radnor, PA). At the time of assay, the insert was either left in this position (conventional transendothelial migration) or flipped 180° (inverted (i) transendothelial migration).

For CCL21 coating, 100 μl gelatin containing 500 ng/ml CCL21 was added to the upper surface of transwell inserts with 100% confluence of iSVEC4-10, incubated for 1 hour at 37 °C, then washed three times with migration medium. For S1P coating, 100 μl gelatin containing 100 nM S1P was added to the surface of transwell inserts before iSVEC4-10 seeding. The monolayer permeability was tested by adding to the upper chamber 100 μl 0.068 mg/ml Evans Blue (Sigma-Aldrich) into migration medium with inserts coated with iSVEC, SVEC, iMS1 or MS1 cells layers. Samples were collected from the lower chamber at 0, 1, 2 and 3 hours; and the OD values of the samples were determined with a microplate reader (Biotech, Winooski, VT) at 620 nm.

For cell migration, a total of 3 × 10^5^ CD4 T cells, CD8 T cells, DC, macrophages, or tumor cells were added in a volume of 100 μl (for flow conditions 340 μl) to the upper chamber of a 24-well transwell plate with a 5 μm pore (for leukocytes) or 8 μm pore (for carcinoma cells) in the insert (Corning International, Corning, NY). Lower wells contained various concentrations of chemokines: 500 ng/ml CCL19, 500 ng/ml CCL21, 50 ng/ml IL6, 200 ng/ml CCL22, 200 ng/ml CCL5, 100 nM S1P, 100 ng/ml CXCL12 or 100 ng/ml CCL22 in 600 μl (for flow conditions 360 μl) of RPMI 1640/0.5% fatty acid-free BSA (Sigma-Aldrich). For mAb blocking, anti-VCAM-1 was used at 1 μg/ml, 3 μg/ml and 10 μg/ml and anti-VLA-4 was used at 1 μg/ml, 3 μg/ml and 10 μg/ml, as previously described^10^. The number of cells that migrated to the lower well after 4 hours was counted with a hemocytometer. For tumor cell migration, cells were labeled with CFSE and migrated toward chemokines for 16 hours. Cells were then collected from both the lower chamber and trypsinization of the lower surface of the inserts, combined, and counted with flow cytometry. The percentage of migrated cells was calculated as number of collected cells divided by total cell input. Each experiment was performed in triplicate at least three times.

### Statistical Analysis

*In vitro* transwell migration assays were performed at least three times for individual experiments, and results represent mean values of triplicate samples. Affymetix human or mouse Genechip array ST2.0 for hLEC, SVEC4-10 and mLEC were performed in triplicate. Immunohistochemistry and H&E staining were performed at least three times for individual experiments, and 10 fields/slide acquired. All flow cytometry experiments were performed at least three times. Fluorescence images were analyzed with Velocity software for quantification of fluorescence density and co-localizaion of markers by Pearson’s Correlation. Other results were analyzed by GraphPad Prism Software (version 5, GraphPad Software, Inc. La Jolla, CA) and presented as the mean ± SD. Statistical analyses were performed using Student’s *t* test. One-way ANOVA was used for multiple comparisons. *P* < 0.05 was considered statistically significant.

## Electronic supplementary material

all supplementary figures

supplementary table 1
