# Supplementary material for: A robust in vitro model for trans-lymphatic endothelial migration
Source: Sci Rep. 2017 May 9;7:1633. doi: 10.1038/s41598-017-01575-w (PMC5431648; doi:10.1038/s41598-017-01575-w)
Supplement: Supplementary file 1 — all supplementary figures [file 41598_2017_1575_MOESM1_ESM.pdf]

Title:

A robust in vitro model for trans-lymphatic endothelial migration

Authors

Yanbao Xiong [1], C. Colin Brinkman [1], Konrad S Famulski[2], Emmanuel F. Mongodin[3], Colin J. Lord [4], Keli L. Hippen [4], Bruce R. Blazar [4], and Jonathan S. Bromberg [1, 5, 6 *]

[1] Center for Vascular and Inflammatory Diseases, University of Maryland School of Medicine

[2] Alberta Transplant Applied Genomics Centre, University of Alberta

[3] Institute for Genome Sciences, University of Maryland School of Medicine

[4] University of Minnesota Cancer Center and the Department of Pediatrics, Division of Blood and Marrow Transplantation, University of Minnesota, Minneapolis, 55455, USA.

[5] Department of Microbiology and Immunology, University of Maryland School of Medicine

[6] Department of Surgery, University of Maryland School of Medicine, Baltimore, Maryland 21201, USA

Supplementary table 1. Comparison of gene array data for primary LEC and SVEC4-10.

Supplementary Table 2. Significant Probe Set Differences Between Primary LEC and SVEC4-10.

Probe Set ID	p-value	adjusted p-value	Gene Accession	Gene Symbol
17283364	2.37E-09	9.80E-05	NM_001082976	Tc2n
17456934	4.25E-08	0.000879	NM_001252292	Mest
17359344	2.22E-07	0.003062	NM_009848	Entpd1
17304523	3.61E-07	0.003318	NM_138672	Stab1
17279670	4.29E-07	0.003318	NM_175930	Rapgef5
17480880	5.05E-07	0.003318	NM_001008548	Pde2a
17206471	6.27E-07	0.003318	---	---
17334803	6.42E-07	0.003318	NM_028101	Jmjd8
17248426	1.06E-06	0.004887	NM_011412	Slit3
17315133	1.29E-06	0.005351	NM_001277255	Acvrl1
17239113	8.61E-06	0.032359	NM_175155	Sash1
17355565	1.09E-05	0.037647	NM_001171010	Slc14a1
17206479	1.24E-05	0.039357	---	---
17271118	1.35E-05	0.039857	NM_145823	Pitpnc1

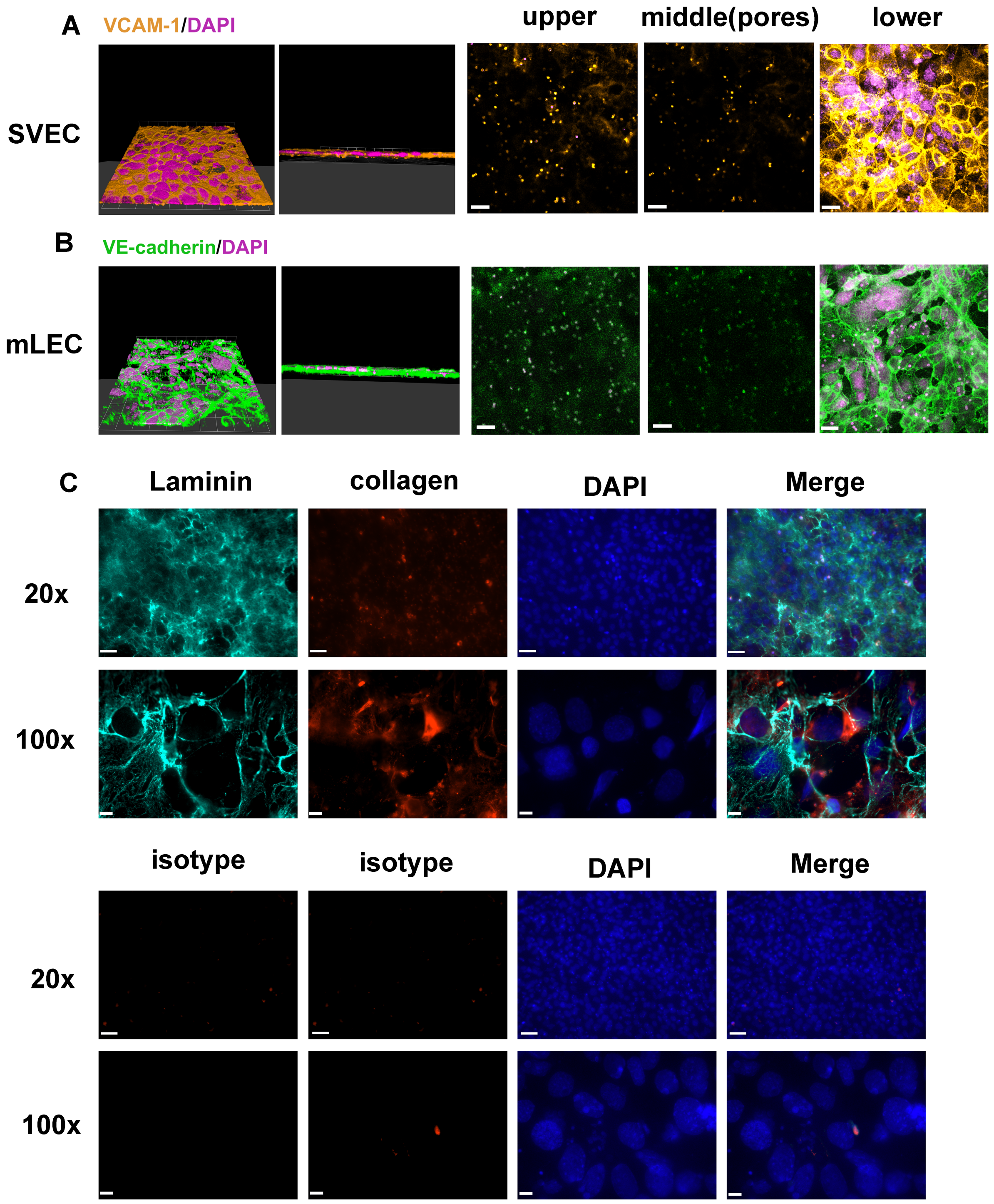

Supplementary Figure 1. (A) SVEC on the inserts were stained with DAPI (violet) and phalloidin (gold) and images were captured with z-stack confocal, ×40, scale bar 21 μm. (B) Mouse LEC on the inserts were stained with DAPI (violet) and anti-VE-cadherin (green) and

images were captured with z-stack confocal imaging, ×40, scale bar 21 µm. (C) SVEC on the inserts were stained with anti-laminin (cyan), anti-collagen (red) and DAPI (blue) and images were captured fluorescence microscopy, ×20, scale bar 42 µm; ×100, scale bar 8 µm. Representative of three independent experiments.

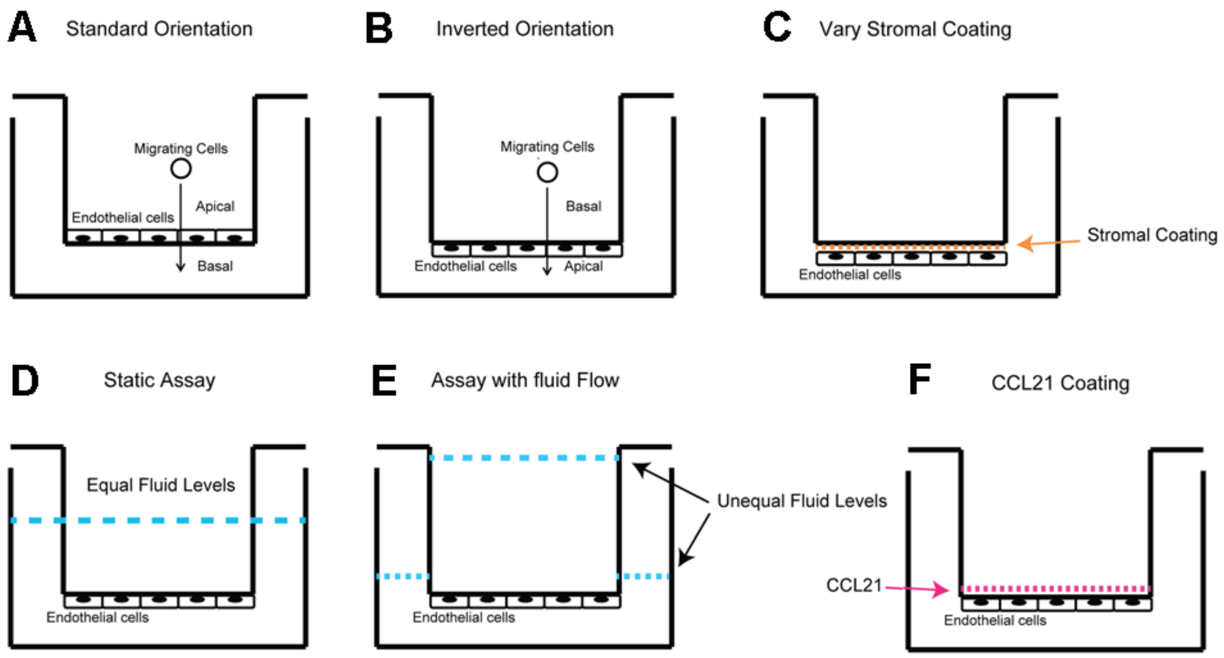

Supplementary Figure 2. Diagram for inserts: (A, B) standard orientation of inserts (SVEC or LEC) and inverted orientation insets (iSVEC or iLEC); (C) inserts with various coating on inserts; (D, E) static assay and fluid flow assay; (F) inserts pre-coated with CCL21.

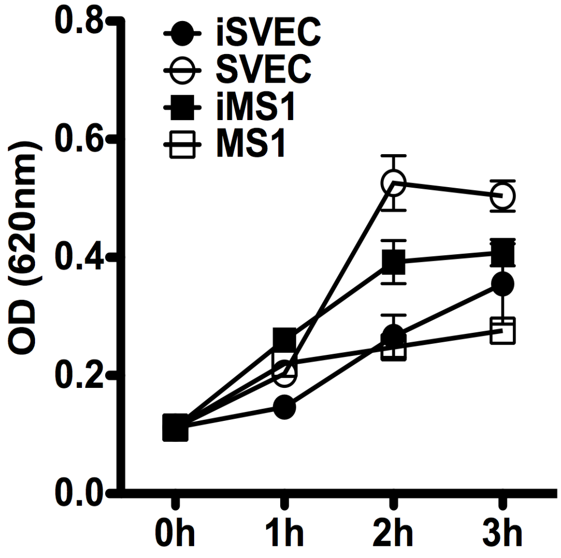

Supplementary Figure 3. Permeability of different monolayers. Evans blue dye added to the upper chamber of the transwell assay with different monolayers coated on the inserts; samples collected at the indicated time points from the lower chamber. Representative of three independent experiments.

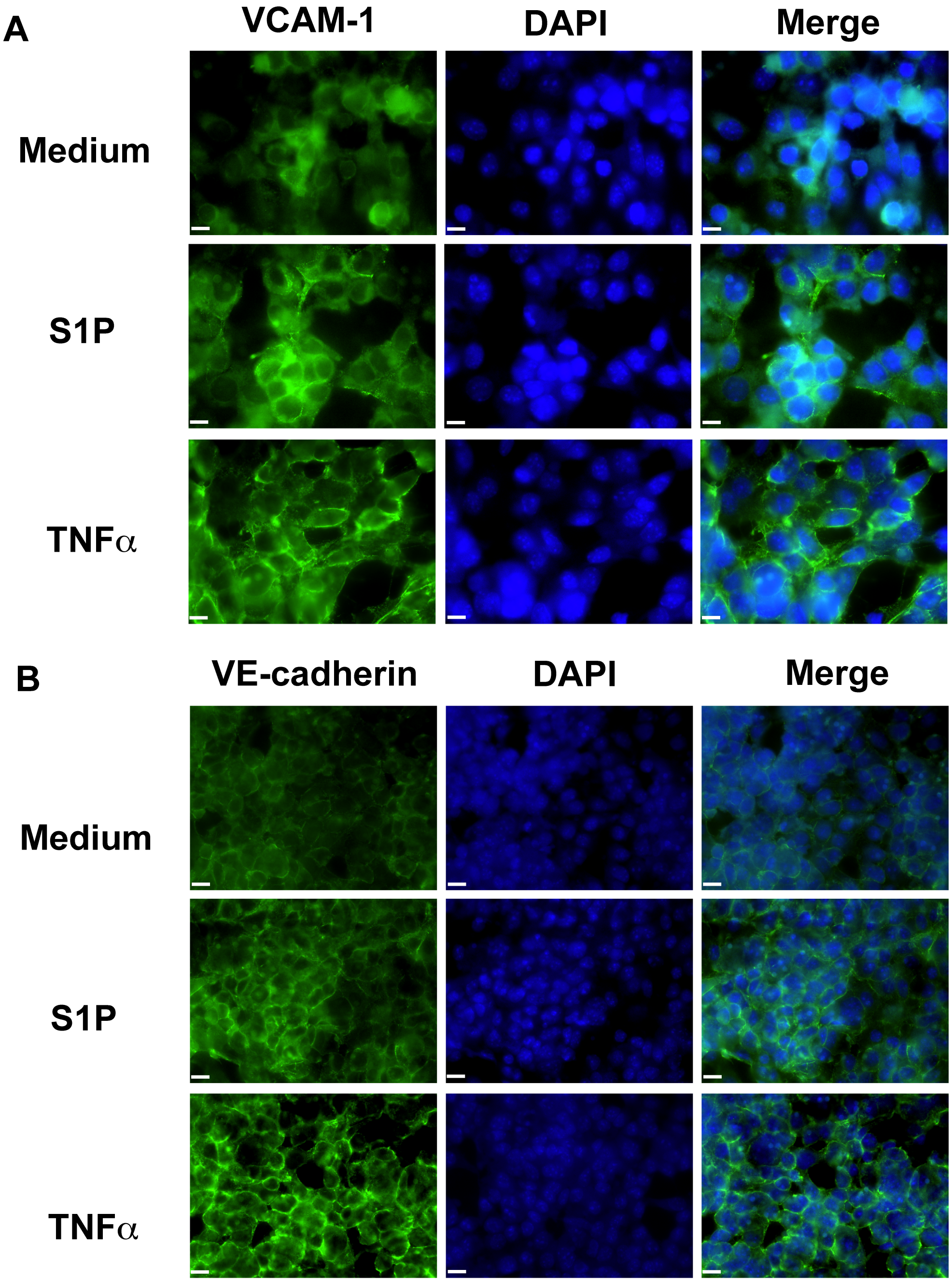

Supplementary Figure 4. (A) SVEC on the inserts were stained with DAPI (blue) and anti-VCAM-1 (green) and images were captured with fluorescence microscopy, × 60, scale bar 14 μm. (B) SVEC on the inserts were stained with DAPI (blue) and anti-VE-cadherin (green) and images were captured with fluorescence microscopy, ×60, scale bar 14 μm. Representative of three independent experiments.

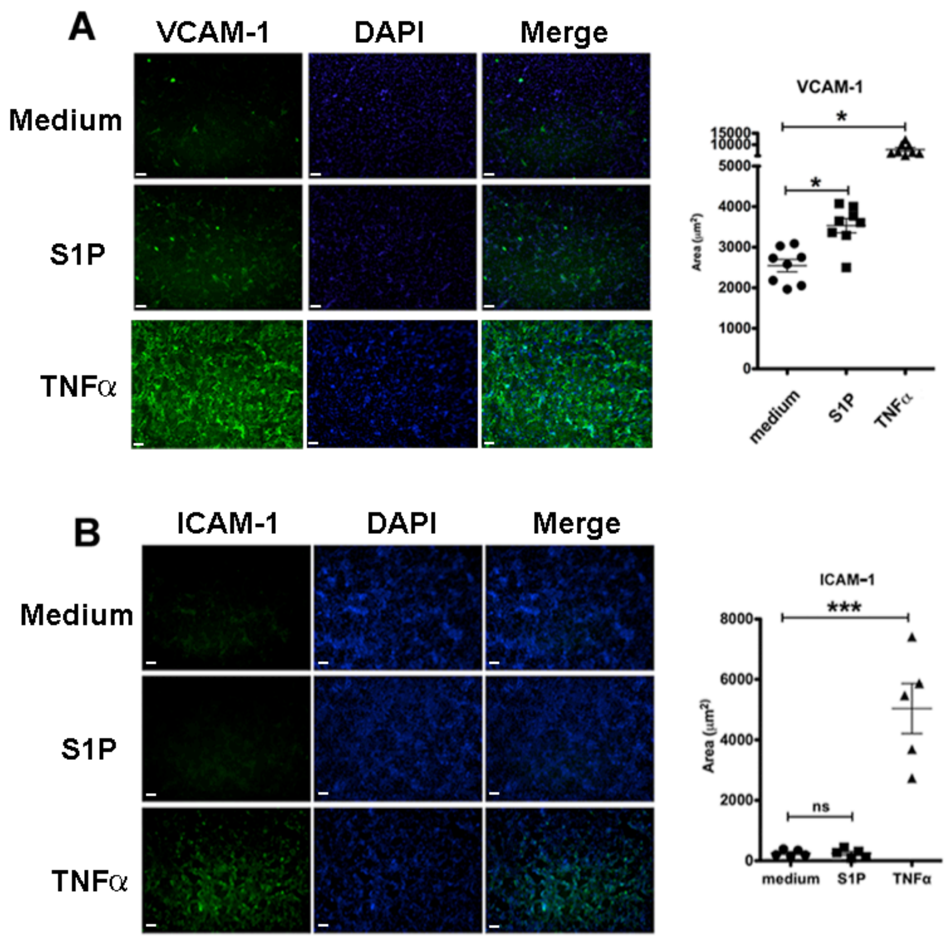

Supplementary Figure 5. Fluorescence staining of LEC layers. (A) VCAM-1 expression on mLEC treated with medium, TNFα or S1P and images were captured with fluorescence microscopy, ×10, scale bar 80 μm. (B) ICAM-1 expression on SVEC4-10 treated with medium, TNFα or S1P and images were captured with fluorescence microscopy, x10, scale bar 80 μm. Representative of three independent experiments. *$P < 0.05$, ***$P < 0.0001$.

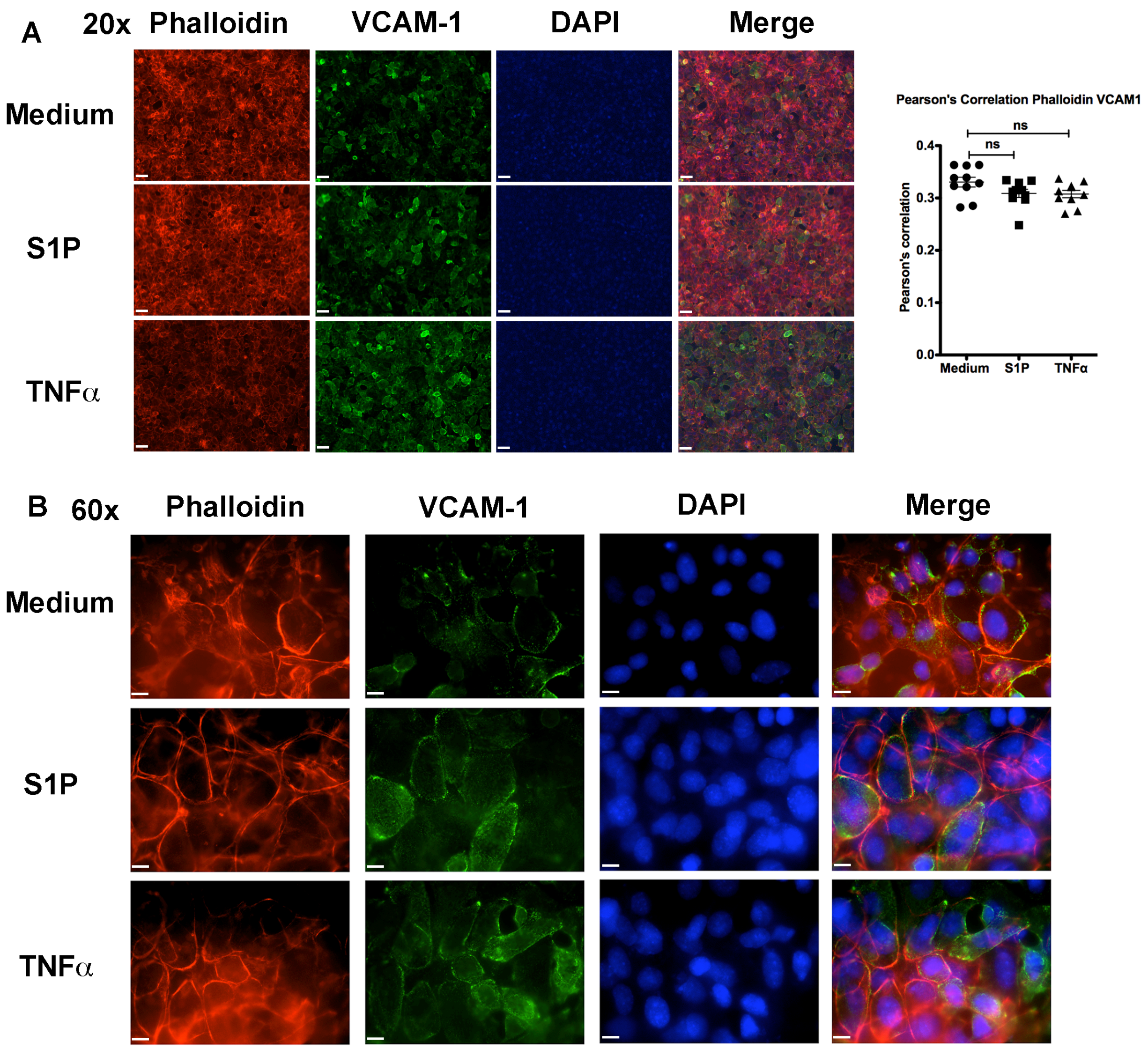

Supplementary Figure 6. (A, B) SVEC on the inserts were stained with DAPI (blue), phalloidin (red) and anti-VCAM-1 (green) and images were captured with fluorescence microscopy, ×20, scale bar 42 μm; ×60, scale bar 14 μm. Representative of three independent experiments.

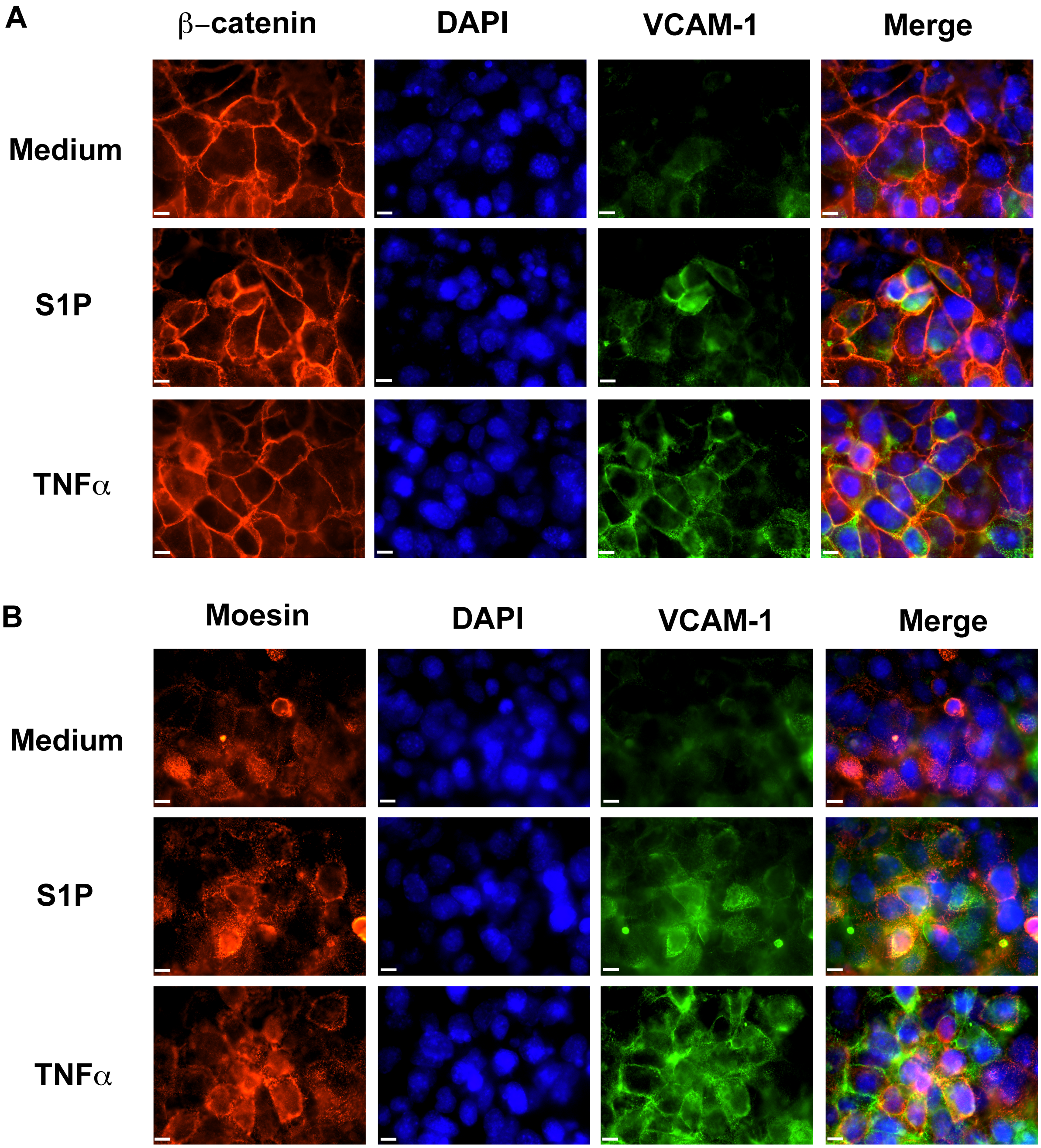

Supplementary Figure 7. (A) SVEC on the inserts were stained with DAPI (blue), anti-β-catenin (red) and anti-VCAM-1 (green) and images were captured with fluorescence microscopy, ×60, scale bar 14 μm. (B) SVEC on the inserts were stained with DAPI (blue), anti-moesin (red) and anti-VCAM-1 (green) and images were captured with fluorescence microscopy, × 60, scale bar 14 μm. Representative of three independent experiments.

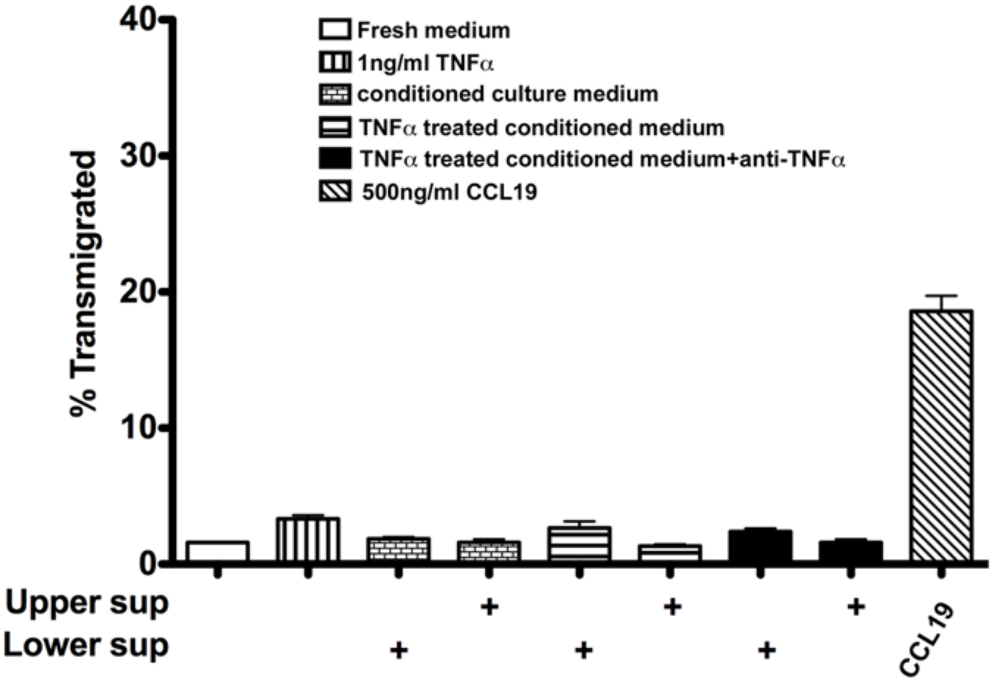

Supplementary Figure 8. LEC conditioned medium does not induce CD4 T cell migration. CD4 T cell migration toward LEC conditioned medium or CCL19. Representative of three independent experiments.
